# Early life stress and hormonal status influence orexin‐1 receptor expression in structures regulating cardiorespiratory responses to CO_2_

**DOI:** 10.1113/EP092431

**Published:** 2025-03-03

**Authors:** Stéphanie Fournier, Julie Plamondon, Denis Richard, Richard Kinkead

**Affiliations:** ^1^ Research Center of the Québec Heart and Lung Institute Laval University Québec City Quebec Canada

**Keywords:** CO_2_ response, control of breathing, orexin, sex based‐differences, stress

## Abstract

Excessive cardiorespiratory responses to CO_2_ are a hallmark of panic disorder (PD). Female sex and exposure to early life stress are risk factors for PD. Neonatal maternal separation (NMS; 3 h/day, postnatal days 3–12) augments the ventilatory response to CO_2_ by ∼35% relative to controls; this effect is most notable during pro‐oestrus but is not observed in males. Orexin‐1 receptor (OX1‐R) antagonism attenuates the CO_2_ response of NMS females. In the limbic system, stress and ovarian hormones influence OX1‐R expression, but the impact of these factors on OX1‐Rs in regions regulating the cardiorespiratory responses to CO_2_ is unknown. Here, we hypothesised that ovarian hormones and NMS determine OX1‐R expression in structures regulating the CO_2_ response; we used in situ hybridisation to quantify OX‐1R mRNA expression in the brains of adult NMS and control rats. Brains were harvested from females that were either in pro‐oestrus (high ovarian hormones) or 2 weeks post ovariectomy (OVX; low ovarian hormones); males were included for comparison. Hormonal status influenced the intensity of the OX1‐R signal in the medial amygdala, raphe obscurus (RObs) and the A5 area, but the direction of the changes (increase vs. decrease) was structure‐specific. Significant NMS × hormonal status interactions were noted in the dorsal raphe, the locus coeruleus, the nucleus of the solitary tract and the A5 area; the effects were structure‐specific. As the dorsal raphe was the only structure in which the changes in OX1‐R expression matched the sex‐specific effect of NMS on the CO_2_ response, this structure likely contributes to respiratory manifestations of PD.

## Abstract (highlights)

- **What is the central question of this study?**
  Anomalies in orexin signalling are an important mechanism of excessive cardiorespiratory response to CO_2_, a hallmark of panic disorder (PD): does early life stress and hormonal status (male, female in pro‐oestrus, and ovariectomised females) influence orexin‐1 receptor (OX1‐R) expression in brain regions regulating the cardiorespiratory responses to CO_2_?
- **What is the main finding and its importance?**
  The effects of stress and hormonal status are structure‐specific. Because the changes in OX1‐Rs observed in the dorsal raphe nucleus match the sex‐specific increase in CO_2_ response, this structure emerges as a primary candidate in the aetiology of PD.

## 1 INTRODUCTION

Orexins A and B (ORX; also known as hypocretins) are regulatory peptides produced by neurons located in the lateral and dorso‐medial/perifornical hypothalamus. Owing to their extensive projections, ORX neurons exert significant influence on brain structures regulating vital functions, including arousal states, food intake and cardiorespiratory homeostasis (Johnson et al., 2010; Nattie & Li, 2012). In turn, sensory signals related to those systems project onto ORX neurons to ensure that their activity matches the physiological needs and psychological states of the organism (Maruyama & Ueta, 2023). However, prolonged or excessive exposure to stressful stimuli increases the risk of neurological diseases such as panic disorder (PD) and post‐traumatic stress disorder (PTSD). Although these pathologies result from different stressors, they share common elements. In both cases, patients are anxious and ∼2 times more likely to suffer from sleep‐disordered breathing (SDB), hypertension and metabolic disorder than the general population (American‐Psychiatric‐Association, 2013; Babson et al., 2013; Berenz et al., 2019; Penninx & Lange, 2018; Zhang et al., 2017). Furthermore, anomalies of the ORX system have emerged as a shared mechanism in the pathophysiology of these disorders (Flores et al., 2015). Remarkably, the prevalence of this condition is ∼2 times higher in women than men (American‐Psychiatric‐Association, 2013; Coryell et al., 2006; Lehner et al., 2021; Leibold et al., 2016; Strawn et al., 2010); yet, the origins of this important sex‐based difference remain poorly understood.

Early life stress is a significant risk factor for PD, and in pre‐clinical research, adult rodents who experienced disruption of maternal care as newborns show key physiological anomalies comparable to those reported in PD, including an elevated hyperventilatory response to CO_2_ (Battaglia et al., 2014). In humans and rodents, excessive behavioural and cardiorespiratory responses to inhalation of a CO_2_‐enriched gas mixture is a reliable biomarker for PD and PTSD (Battaglia & Khan, 2018; Coryell et al., 2006; Gorman et al., 1994; Leibold et al., 2016; Muhtz et al., 2011; Smits et al., 2022). In women with premenstrual symptoms, the panicogenic effects of CO_2_ inhalation are highest during the pre‐menstrual phase, thus indicating that ovarian hormones exacerbate manifestations of the disorder (Nillni et al., 2017). Female rats previously subjected to neonatal maternal separation (NMS; 3 h/day, postnatal days 3–12) show an excessive hyperventilatory response to 5% CO_2_, which is more notable during pro‐oestrus (Battaglia et al., 2014; Genest et al., 2007; Tenorio‐Lopes et al., 2020). This effect of NMS on respiratory control is sex‐specific, as it was not observed in males (Genest et al., 2007). In NMS females, the inhibitory action of a selective orexin‐1 receptor (OX1‐R) antagonist on the CO_2_ response is greatest during pro‐oestrus (Tenorio‐Lopes et al., 2020). Exposure to more severe CO_2_ levels (35%) can induce panic‐related behaviours and cardiovascular responses in unstressed males, which can be attenuated only by OX1‐R antagonism; agents inactivating OX2‐Rs are ineffective (Johnson et al., 2015). Together, these observations are consistent with results showing that in rats, expression of OX1‐Rs in the hypothalamus parallels ovarian hormone levels and peaks during pro‐oestrus (Silveyra et al., 2009), and that in males, exposure to stress (NMS, acute prolonged stress) up‐regulates OX1‐R expression in the prefrontal cortex and hippocampus, respectively (Feng et al., 2007; Han et al., 2020).

Stable matching of neurotransmitters with their receptors is fundamental to synapse function and efficient communication in neural circuits (Godavarthi et al., 2024). Typically, presynaptic neurotransmitters regulate expression of postsynaptic receptors and although dynamic changes in OX1‐R expression may well be an important mechanism in the pathophysiology of stress‐related neurological disorders, the impacts of stress and hormonal status on OX1‐R expression have been evaluated separately. Furthermore, the current literature has focused mainly on tissue homogenates from the hypothalamus and hippocampus. Although physiological symptoms are important in PD and PTSD, there is limited information (if any) on expression of OX1‐Rs in regions associated with cardiorespiratory homeostasis, especially in females. As this knowledge is essential to understand the aetiology of stress‐related disorders, we tested the hypothesis that ovarian hormones and NMS interact to determine OX1‐R expression in neural structures regulating the reflexive responses to CO_2_. We used in situ hybridisation as this technique allows quantification of OX‐1R with good anatomical precision. Based on the pattern of NMS‐ and sex‐related changes in OX1‐R expression, our secondary goal was to identify candidate structures in which anomalies in ORX signalling contribute to stress‐induced enhancement of the CO_2_‐response.

## 2 METHODS

### 2.1 Ethical approval and animals

Experiments were performed on sexually mature male (*n* = 16) and female (*n* = 32) Sprague–Dawley rats. All animals were born and raised in the animal care facilities of the Research Center of the Québec Heart & Lung Institute (Québec, QC, Canada). The adult males (200–225 g) and females (180–200 g) used for mating were obtained from Charles River Canada (St‐Constant, QC, Canada). Rats were supplied with food and water ad libitum and maintained in standard conditions (21°C, 12:12‐h dark–light cycle: lights on at 06.00 h and off at 18.00 h). The Animal Care Committee of Université Laval approved all the experimental procedures and protocols (Protocol no. 16–020), which were in accordance with the ARRIVE (2.0) guidelines and the guidelines of the Canadian Council on Animal Care. All personnel involved in this research followed appropriate training and complied with the institutional and national ethical guidelines, which are consistent with the standards of *Experimental Physiology*.

### 2.2 Neonatal maternal separation (NMS)

The NMS protocol was identical to the one used in our previous studies (Genest et al., 2007; Tenorio‐Lopes et al., 2020). Briefly, sexually mature virgin females were mated, and 3 days after delivery, each litter was separated daily from their mother 3 h/day (09.00–12.00 h) from post‐natal day 3 to 12. Pups maintained under standard rearing conditions over that period were used as controls as this treatment is considered the most appropriate reference group for studies investigating the effects of maternal separation (Lehmann & Feldon, 2000) (see Gulemetova & Kinkead, 2011 for more detailed discussion). All rats were weaned at P21; they received standard animal care and were raised in the animal care facilities of the Research Center of the Québec Heart & Lung Institute until experiments were performed when rats reached 7–10 weeks of age. For each group, rats originated from at least three different litters to avoid litter‐specific effects.

### 2.3 Hormonal status: rationale and procedures

As PD and PTSD are more prevalent in women than men, our main interest was to evaluate the impact of endogenous ovarian hormones on OX1‐R expression. To do so, we first harvested brains from two groups of females: during pro‐oestrus (when ovarian hormones are naturally elevated) or 2 weeks following ovariectomy (OVX, surgical reduction of ovarian hormones). We also harvested brains from males to evaluate sex‐based differences; however, gonadectomy was not performed.

#### 2.3.1 Ovariectomy (OVX)

According to standard procedures (Fournier et al., 2015; Tenorio‐Lopes et al., 2020), females were anaesthetised with isoflurane (2% in air). Once a surgical plane of anaesthesia was achieved, hydration was ensured by subcutaneous injection of lactated Ringer solution (0.5 mL/100 g). Prior to surgery, analgesia was ensured at the incision site (lidocaïne/bupivacaïne 7 mg/kg s.c.) and systemically (buprenorphine; 0.05 mg/kg s.c.). Then 1−1.5 cm vertical incisions were made in the lower back. The muscle was cut on each flank and a long strip of pale adipose tissue was pulled out of each incision. This tissue links to the fallopian tubes and the ovaries. Ovariectomised rats had both ovaries clamped, sutured and completely removed. Following surgery, analgesia was ensured by subcutaneous injection of meloxicam (1 mg/kg) immediately after surgery and the following day. Rats were observed daily for the first 5 days post‐op. Rats recovered for 2 weeks before experiments were performed. Data from OVX females were compared to those obtained in pro‐oestrus; sham‐surgeries were not performed because previous work did not reveal physiological or endocrine differences between sham‐operated and non‐operated females (Fournier et al., 2015; Tenorio‐Lopes et al., 2020). In line with ethical recommendations, this approach reduced the number of animals used and avoided unnecessary procedures to the animals.

#### 2.3.2 Identification of the oestrous cycle

The oestrous cycle was identified by vaginal smear, as done previously (Tenorio‐Lopes et al., 2020). As NMS increases vaginal sensitivity (Pierce et al., 2014), multiple vaginal smear sampling was avoided. To increase the probability of harvesting brains at the desired phase, a vaginal smear was performed 3–4 days before the experiments and the time when the female would be in pro‐oestrus was estimated; with this approach, ∼75% of the females were in pro‐oestrus when brains were harvested. Final identification of the oestrous cycle was performed when rats were deeply anaesthetised for brain harvesting to avoid potential stress to the rat. Brains harvested from females that were not in pro‐oestrus were not included in this study; they were stored and ultimately assigned to other projects.

### 2.4 Quantification of OX1‐Rs with in situ hybridisation

#### 2.4.1 Collection of brain tissue and processing

Circadian rhythm impacts the ORX system, and its influence on the ventilatory control and cyclic changes in OX1‐R expression contributes to this effect (Dias et al., 2010; Silveyra et al., 2009). Accordingly, all brains and blood were collected between 09.00 and 12.30 h. Rats were deeply anaesthetised with an intraperitoneal injection of a mixture of ketamine (80 mg/kg) and xylazine (10 mg/kg); body weight was measured, and a cardiac puncture was performed to collect blood for analysis of plasma levels of ORX_A_ (see details below). Rats were then perfused with 0.9% saline followed by 4% paraformaldehyde (PFA) in 0.1 M sodium tetraborate buffer (PFA–borax; pH 9.5 at 4°C). Brains were removed from the skull, post‐fixed for 2 weeks in 4% PFA–borax, and then placed in 20% sucrose–4% paraformaldehyde solution for 48 h at 4°C. Frozen brains were mounted on a microtome and then cut into 30 µm coronal sections. Slices were collected in a cold cryoprotectant solution and stored at –20°C until experimentation.

#### 2.4.2 In situ hybridisation

In situ hybridisation histochemistry was used to localise OX1‐R mRNAs on tissue sections taken from the rat brain. The protocol used was largely adapted from the technique described by Simmons et al. (1989). Briefly, the brain sections were mounted onto slides and allowed to desiccate overnight under a vacuum. They were then successively fixed, digested, and dehydrated through graded concentrations of alcohol. After vacuum drying, 100 µL of the hybridisation mixture, which contains an antisense ^35^S‐labelled cRNA probe (107 cpm/mL), was spotted on each slide. The slides were sealed under a coverslip and incubated. The next day, coverslips were removed, and the slides were rinsed, digested, washed and dehydrated. After vacuum drying, the slides were exposed to an X‐ray film (Eastman Kodak, Rochester, NY, USA). Once removed from the autoradiography cassettes, the slides were defatted in xylene and dipped in NTB2 nuclear emulsion (Eastman Kodak). Because signal intensity varies substantially between brain regions, exposure times were adjusted to optimise the signal. Slides containing brainstem sections were exposed for 6 weeks; slides containing the locus coeruleus (LC) were exposed for 2 weeks, and slides containing the hypothalamus were exposed for 5 weeks. Slides were then developed in D19 developer (Eastman Kodak) and fixed in rapid fixer (Eastman Kodak) for 5 min. Finally, tissues were rinsed, counterstained with thionin (0.25%), dehydrated, cleared in xylene and coverslipped with Dibutylphthalate Polystyrene Xylene (DPX).

#### 2.4.3 ^35^S‐labelled cRNA probe

We generated an OX1‐R cRNA probe from 1910–2169 bp of the rat OX1‐R cDNA, gene bank accession number AF041244. The 400‐bp fragment of OX1‐R cDNA was produced by RT‐PCR from cDNA derived from total rat brain RNA. Our probe included the coding sequence for the complete I and II hydrophobic domains, complete II hydrophilic domains and partial I and III hydrophilic domains of the OX1‐R protein. The identity of the cloned fragment was verified by sequencing. The PCR product of OX1‐R cDNA was subcloned into pGEM‐T plasmid (Promega, Madison, WI, USA), which was linearised with *Spe*1 and *Aat*II endonucleases for the antisense and sense probes, respectively. The radioactive riboprobe was synthesised by incubation of 250 ng of linearised plasmid in 10 mM NaCl, 10 mM dithiothreitol, 6 mM MgCl_2_, 40 mM Tris (pH 7.9), 0.2 mM ATP/GTP/CTP, 100 µCi of a‐35S‐UTP, 40 U RNasin (Promega) and 20 U of Sp6 and T7 RNA polymerase for sense and antisense probes, respectively, for 60 min at 37°C. The DNA templates were treated with 100 µL of DNAse solution (1 µL DNAse, 5 µL of 5 mg/mL tRNA, 94 µL of 10 mM Tris/10 mM MgCl_2_). The purification of the riboprobes was accomplished by using a Qiagen RNeasy mini kit (Qiagen, Mississauga, ON, Canada). The specificity of the probe was confirmed by the absence of positive signal in hybridised sections with sense probe.

### 2.5 Quantitative analysis of the hybridisation signal

The hybridisation signal revealed on NTB2‐dipped nuclear emulsion slides was analysed and quantified under a light microscope (Olympus BX51 60; Olympus, Tokyo, Japan) equipped with a video camera (Evolution QEi camera, Media Cybernetics Inc, Bethesda, MD, USA) coupled to a PC using Image Pro‐Plus software, version 7.0 (Media Cybernetics). The intensity of the hybridisation signal was measured under darkfield illumination at a magnification of ×4. Saturation of the hybridisation signal was avoided by adjusting the exposure time for the image with the strongest hybridisation signal sampled for each region. The luminosity of the system was set to the maximum, and the saturation warning option was used to visualise saturated regions in the image preview. Thereafter, according to the pixel distribution histogram, the exposure time was adjusted to reduce the number of saturated (pure white) pixels to zero. The same luminosity and exposure time were conserved for the analysis of the entire series.

The analyses focused on structures associated with the reflexive cardiorespiratory and behavioural responses to CO_2_ (Bernabe et al., 2024; Schenberg, 2016). To ensure reliable quantification, only structures with robust OX1‐R signals in all groups were considered. These include the LC (9.8–10.04 mm caudal to the bregma) (Paxinos & Watson, 1998); the medial part of the nucleus tractus solitarius (NTS; 13.24–13.30 mm caudal to the bregma) (Paxinos & Watson, 1998); the dorsal, ventral and ventrolateral divisions of the dorsal raphe (DRD, DRV, and DRVL, respectively; 8.00–8.30 mm caudal to the bregma) (Paxinos & Watson, 1998); the noradrenergic A5 region (A5; 10.30–10.52 mm caudal to the bregma) (Paxinos & Watson, 1998); The central, medial and basolateral amygdala (CeA, MeA, and BLA, respectively; 2.8‐3.14 mm caudal to bregma) (Paxinos and Watson, 1998); and the raphe obscurus (RObs) (13.24–13.30 mm caudal to the bregma) (Paxinos & Watson, 1998). These regions were outlined, and the measurements of the optical density (OD) of the hybridisation signal were performed separately on each side of the brain on the two to four sections of each animal assigned to each treatment. The surface area of the region identified under the dark field was traced and measured using the microscope's image analysis software (ImageJ; National Institute of Health, Bethesda, MD, USA). Statistical analyses confirmed that none of the experimental factors (NMS and hormonal status) affected the surface area. When the hybridisation signal was less visible under darkfield illumination, the brain structures of interest (BLA, MeA and dorsal raphe (DR)) were outlined under brightfield illumination and then subjected to densitometric analysis under darkfield illumination. The OD for each specific region was corrected for the average background signal, which was determined by sampling unlabelled areas outside of the areas of interest.

### 2.6 Quantification of plasma ORX_A_ levels

Quantification of ORX_A_ levels in the cerebrospinal fluid (CSF) or hypothalamic tissue extracts is a common approach to evaluate the functionality of ORX neurons; in humans, measurements of plasma levels nonetheless offer a reliable and less invasive alternative (Zhu et al., 2020). Blood samples obtained by cardiac puncture were placed in an Eppendorf tube containing EDTA with aprotinin (0.6 TIU/mL of blood); it was then centrifuged, and the plasma was stored at −80°C until quantification by commercially available enzyme immunoassay kit for orexin‐A (Phoenix Pharmaceuticals Inc, Burlingame, CA, USA); the kit's sensitivity is 0.22 ng/mL. Samples were read on a microplate spectrophotometer (Quant; Bio‐Tek Instruments Inc., Winooski, VT, USA). ORX_A_ concentrations were calculated from the four‐parameter logistic standard curve using SigmaPlot 13 (Systat Software, San Jose, CA, USA).

### 2.7 Statistics

Multifactorial analysis of variance (ANOVA) assessed the effects of NMS and hormonal status (pro‐oestrus, OVX, male) on the OD of the hybridisation signal; equality of variance was tested. ANOVA results are reported in the figures for clarity and conciseness. Individual data are reported in the figures; histogram height represents the mean of the OD signal. Error bars indicate the standard deviation. No animals or data points were excluded from the analyses. A significant ANOVA result (*P* ≤ 0.05) was followed by a *post hoc* test (Fisher's least significant difference) to identify specific differences. Comparisons were conditional on each factor to reduce the number of comparisons and a Bonferroni correction was applied. As ANOVA did not reveal a significant effect of NMS alone in any of the structures tested, *post hoc* testing for differences between NMS and controls was performed only when a factorial interaction was significant (NMS × hormonal status). Note that results from ANOVAs and *post hoc* tests are reported only in the figures to avoid overloading the text. Analyses were performed using JASP (version 0.19.1; University of the Netherlands).

## 3 RESULTS

### 3.1 Body weight as an indicator of food intake and metabolism

Overall, OVX females weighed more than females with intact gonads, and males weighed more than either group of females. The effect of NMS on body weight was sex‐specific. Males subjected to NMS weighed 24% more than controls; NMS did not affect females’ body weights (Table 1).

**TABLE 1:** Effect of NMS on the body weight of male and female rats.

	Control			NMS					
	Females pro‐oestrus (*n* = 8)	Females OVX (*n* = 8)	Male (*n* = 8)	Females pro‐oestrus (*n* = 8)	Females OVX (*n* = 8)	Male (*n* = 8)	NMS effect	Hormonal status effect	Interaction
Body weight (g)	294 ± 31***	379 ± 30‡	392 ± 21	279 ± 67***	345 ± 42***‡‡	488 ± 69†††	*P* = 0.262 *F*_(1, 42)_ = 0.13	*P* < 0.001 *F*_(2, 42)_ = 42.36	*P *< 0.001 *F*_(2, 42)_ = 8.72

*Note*: In females, the effects of ovarian hormones were assessed by comparing the weights of females with intact ovaries during pro‐oestrus with that of rats in which ovaries were removed surgically (ovariectomy; OVX) 2 weeks prior to experimentation; males were included for comparison, but gonadectomy was not performed. Values are reported as means ± SD. Within each group, ***indicates a value significantly different from corresponding male values at *P* < 0.001; ‡significantly different from corresponding pro‐oestrus value at *P* < 0.05; ‡‡significantly different from pro‐oestrus at *P* < 0.01; †††significantly different from corresponding control value at *P* < 0.001. Abbreviations: NMS, neonatal maternal separation; OVX, ovariectomy.

### 3.2 OX1‐R expression in the amygdala

**FIGURE 1:**
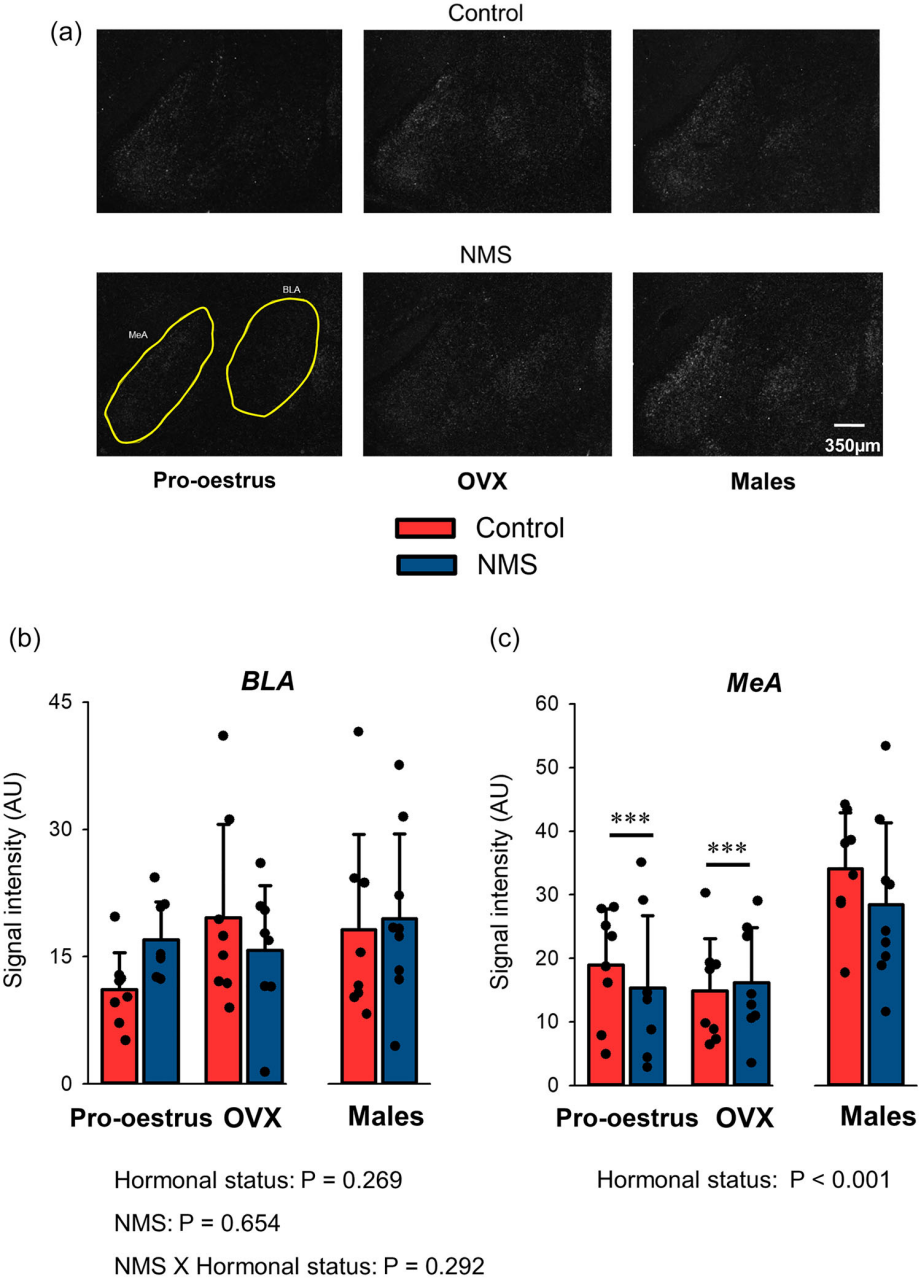
Effects of hormonal status and NMS on OX1‐R expression in the main subregions of the amygdalar complex. (a) Representative photomicrographs comparing the intensity of the OX1‐R mRNA signal obtained by in situ hybridisation in the BLA and the MeA. The yellow areas indicate the subregions analysed in each image; it was not repeated on all panels for simplicity. (b, c) Group data in the BLA (b) and the MeA (c). In each graph, bars represent the mean ± SD; each data point represents the value from a single animal. ***Significantly different from corresponding males at *P* < 0.001. BLA, basolateral amygdala; MeA, medial amygdala; NMS, neonatal maternal separation; OX1‐R, orexin‐1 receptor.

The amygdalar complex is composed of multiple subregions; however, only the medial and basolateral regions of the amygdala (MeA and BLA) were analysed as the signal observed in other areas (e.g., the central amygdala) was too weak and/or variable for reliable quantification. Whereas OX1‐R expression in the BLA was unaffected by the experimental factors, the intensity of the OX1‐R signal measured in the MeA was ∼2 times greater in males than females, regardless of ovarian function (Figure 1). Table 2 reports a summary of the main results obtained in the amygdala and other structures.

**TABLE 2:** Summary of the effects of hormonal status and NMS on OX1‐R expression in selected brain regions regulating cardiorespiratory responses to CO_2_.

Structure	Group	Pro‐oestrus	OVX	Male
BLA	Control	+	+	+
	NMS			
MeA	Control	+	+	++
	NMS			
LC	Control	+++	++	+++
	NMS	++	+++	++
DRVL	Control	+	++	++
	NMS	++	++	++
DRD	Control	+	++	++
	NMS	++	++	++
DRV	Control	+	++	++
	NMS	++	++	++
RObs	Control	+++	++	++
	NMS			
NTS	Control	+	+	++
	NMS	+	++	+
A5	Control	+	+	++
	NMS	+	++	++
Plasma orexin	Control	+	+	++
	NMS			

*Note*: The effects of ovarian hormones were assessed by comparing females with intact ovaries during pro‐oestrus with ovariectomised females (OVX); males were included for comparison, but gonadectomy was not performed. In each structure, the number of ‘+’ indicates the relative intensity of the OX1‐R signal. Note that data for NMS and controls were pooled when ANOVA showed no significant effect of NMS or NMS × hormonal status interaction. Symbols in blue indicate a value significantly different from corresponding pro‐oestrus value (*P* < 0.05); symbol in red indicate a value significantly different from corresponding control value (*P* < 0.05). See text and figures for more details. Abbreviations: BLA, basolateral amygdala; DR, dorsal raphe; DRD, dorsal subregion of the DR; DRV, ventral subregion of the DR; DRVL, ventrolateral subregion of the DR; LC, locus coeruleus; MeA, medial amygdala; NMS, neonatal maternal separation; NTS, nucleus of solitary tract; OX1‐R, orexin‐1 receptor; RObs, raphe obscurus.

### 3.3 OX1‐R expression in the midbrain and pons

#### 3.3.1 OX1‐R expression in the LC

**FIGURE 2:**
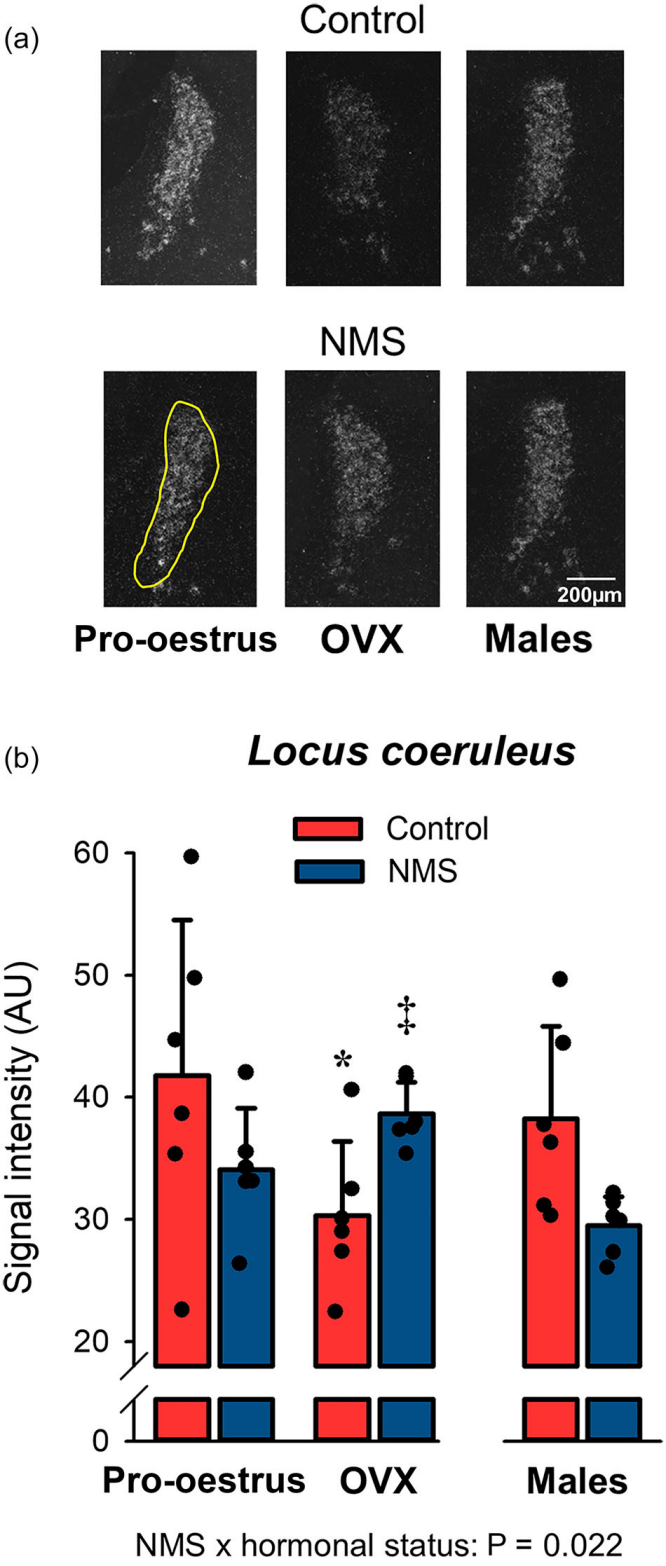
Effects of hormonal status and NMS on OX1‐R expression in the LC. (a) Representative photomicrographs comparing the intensity of the OX1‐R mRNA signal obtained by in situ hybridisation in the LC. The yellow area indicates the region analysed in each image; it was not repeated on all panels for simplicity. (b) In this graph, bars represent the mean ± SD; each data point represents the value from a single animal. *Different from corresponding pro‐oestrus value at *P* < 0.05. ‡Different from corresponding control value at *P* < 0.06. LC, locus coeruleus; NMS, neonatal maternal separation; OX1‐R, orexin‐1 receptor.

The LC had the highest signal intensity of all the structures studied. OVX reduced OX1‐R expression in controls but not NMS. Following OVX, ORX1‐R expression was 27% higher in NMS than controls (Figure 2; Table 2).

#### 3.3.2 OX1‐R expression in the DR

**FIGURE 3:**
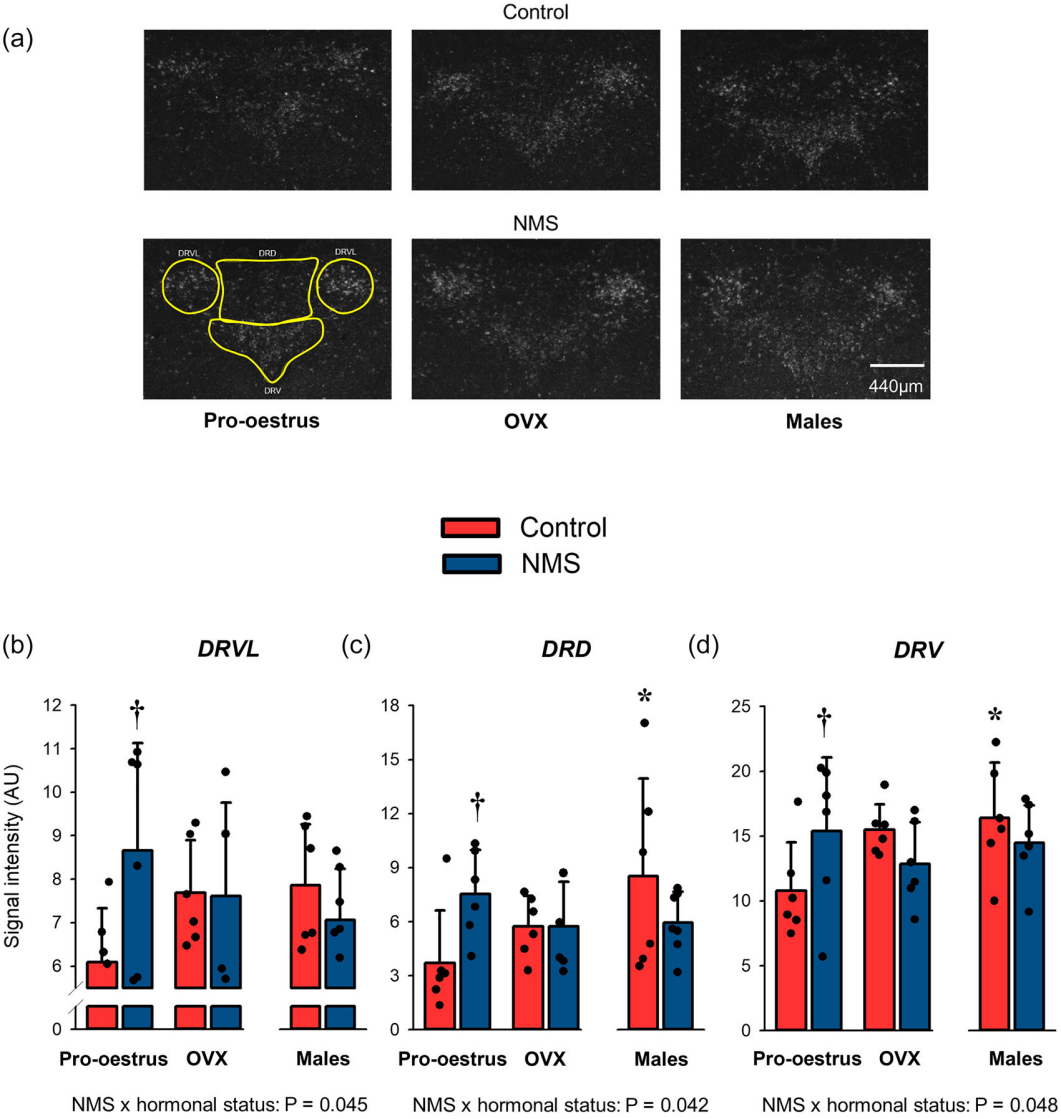
Effects of hormonal status and NMS on OX1‐R expression in the main subregions of the DR. (a) Representative photomicrographs comparing the intensity of the OX1‐R mRNA signal obtained by in situ hybridisation in the DRVL, the DRD and the DRV. The yellow areas indicate the subregions analysed in each image; it was not repeated on all panels for simplicity. (b–d) Group data in the DRVL (b), the DRD (c), and the DRV (d). In each graph, the bars represent the mean ± SD; each data point represents the value from a single animal. **†**Different from corresponding control value at *P* < 0.05. *Different from corresponding pro‐oestrus value at *P* < 0.05. DR, dorsal raphe; DRD, dorsal subregion of the DR; DRV, ventral subregion of the DR; DRVL, ventrolateral subregion of the DR; NMS, neonatal maternal separation; OX1‐R, orexin‐1 receptor.

Here, NMS and hormonal status interacted significantly to influence OX1‐R expression. The effects were similar in the three main DR subdivisions analysed; in controls, the lowest OX1‐R expression was observed in females with the highest levels of ovarian hormones (pro‐oestrus) and the most intense signal was observed in males (Figure 3; Table 2). However, NMS reversed this trend as the highest OX1‐R expression was observed in females exposed to NMS measured in pro‐oestrus.

### 3.4 OX1‐R expression in the medulla

#### 3.4.1 RObs: OX1‐R expression is highest in pro‐oestrus

**FIGURE 4:**
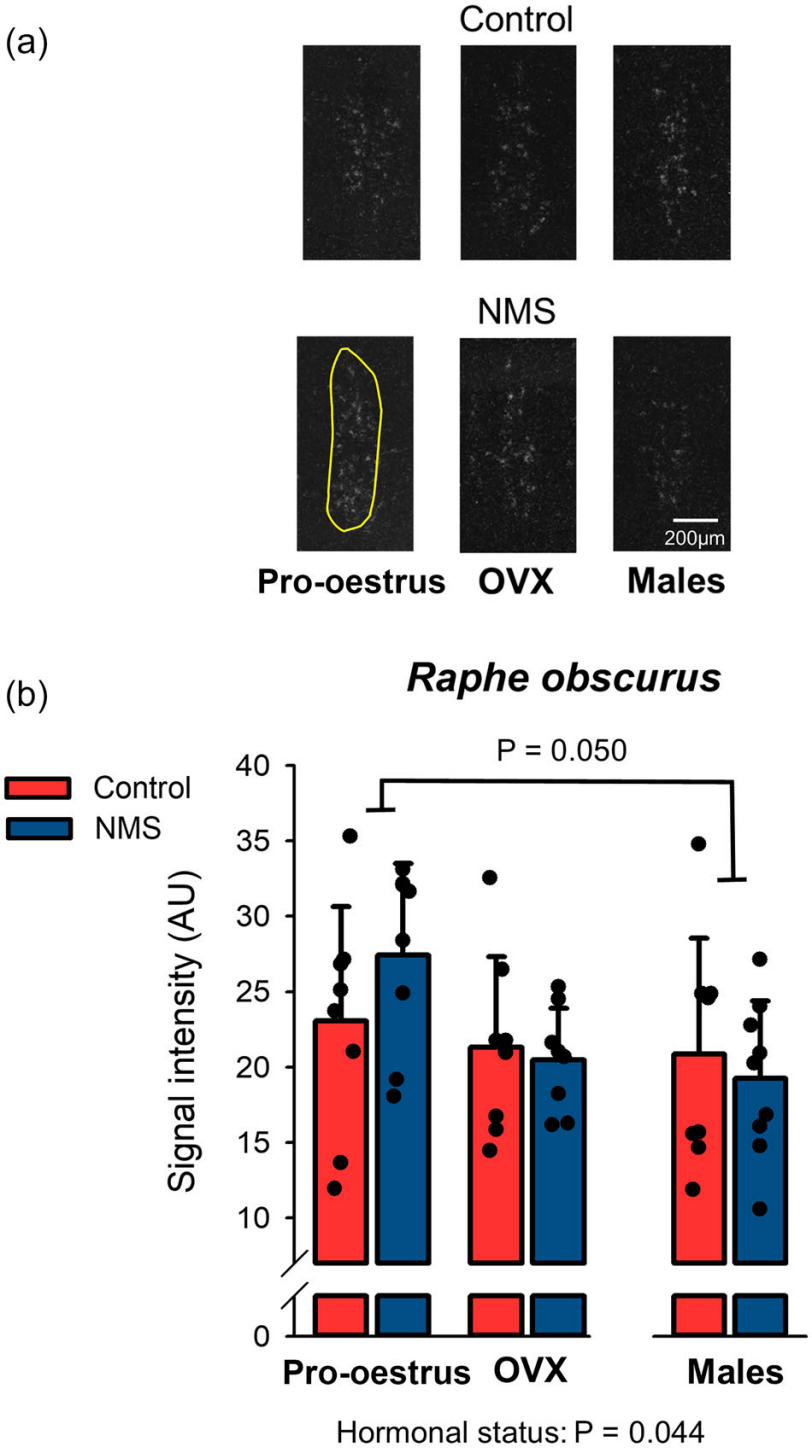
Effects of hormonal status and NMS on OX1‐R expression in the RObs. (a) Representative photomicrographs comparing the intensity of the OX1‐R mRNA signal obtained by in situ hybridisation in the RObs. The yellow area indicates the region analysed in each image; it was not repeated on all panels for simplicity. (b) In this graph, bars represent the mean ± SD; each data point represents the value from a single animal. NMS, neonatal maternal separation; OX1‐R, orexin‐1 receptor; RObs, raphe obscurus.

OX1‐R expression in the RObs was influenced by hormonal status but not NMS. The signal intensity measured in females during pro‐oestrus was 26% greater than that of males (Figure 4; Table 2).

#### 3.4.2 OX1‐R expression in the nucleus tractus solitarius

**FIGURE 5:**
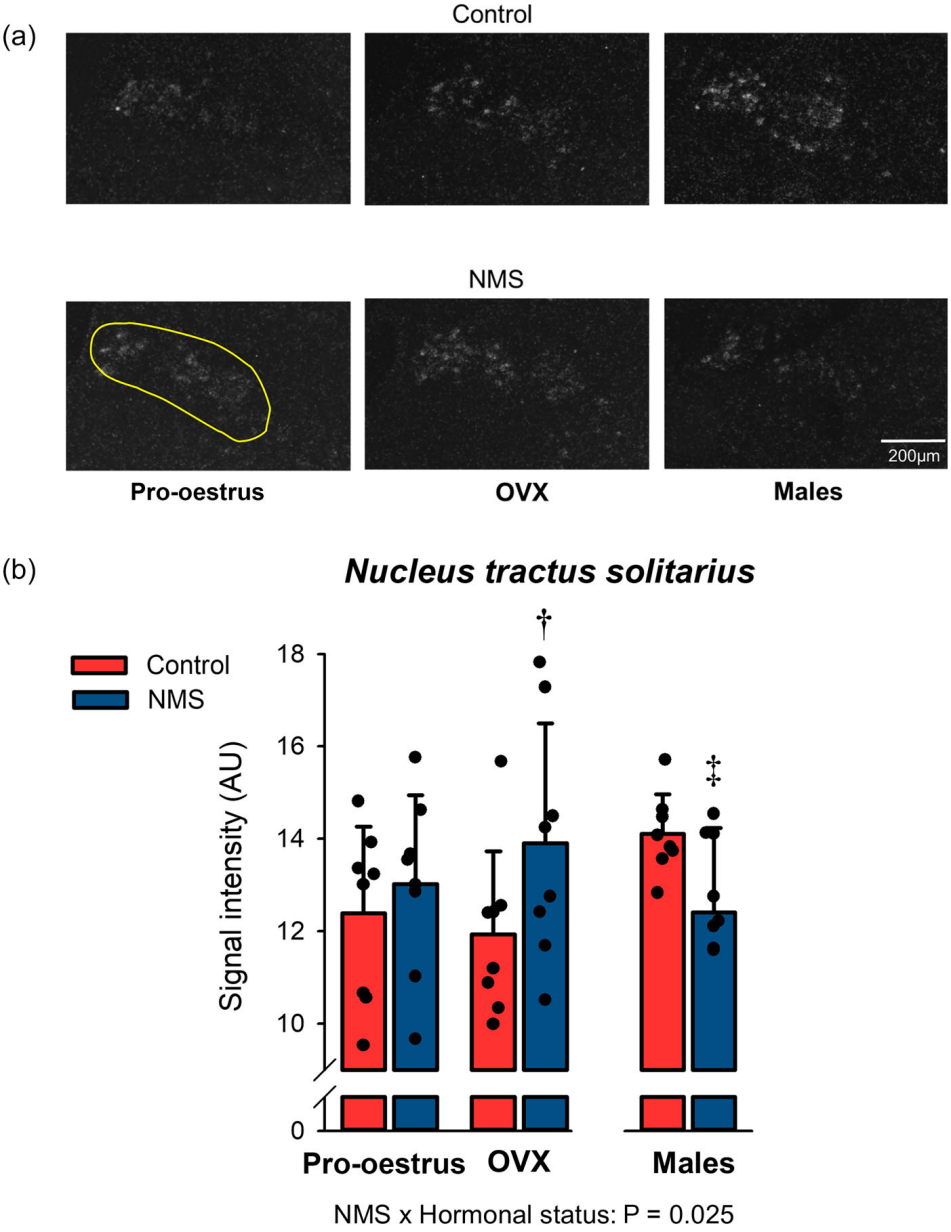
Effects of hormonal status and NMS on OX1‐R expression in the NTS. (a) Representative photomicrographs comparing the intensity of the OX1‐R mRNA signal obtained by in situ hybridisation in the NTS. The yellow area indicates the region analysed in each image; it was not repeated on all panels for simplicity. (b) In this graph, bars represent the mean ± SD; each data point represents the value from a single animal. **†**Different from corresponding control value at *P* < 0.05. ‡Different from corresponding control value at *P* < 0.07. NMS, neonatal maternal separation; NTS, nucleus of the solitary tract; OX1‐R, orexin‐1 receptor.

In controls, OX1‐R signal intensity was similar in both groups of females but tended to increase in males (OVX vs. males: *P* = 0.077). This was not observed in NMS rats, in which the highest signal intensity was observed in OVX females; this value was 17% greater than in controls. The lowest NMS mean value was observed in males (12 ± 1.8 AU; Figure 5; Table 2).

#### 3.4.3 OX1‐R expression in the noradrenergic A5 area

**FIGURE 6:**
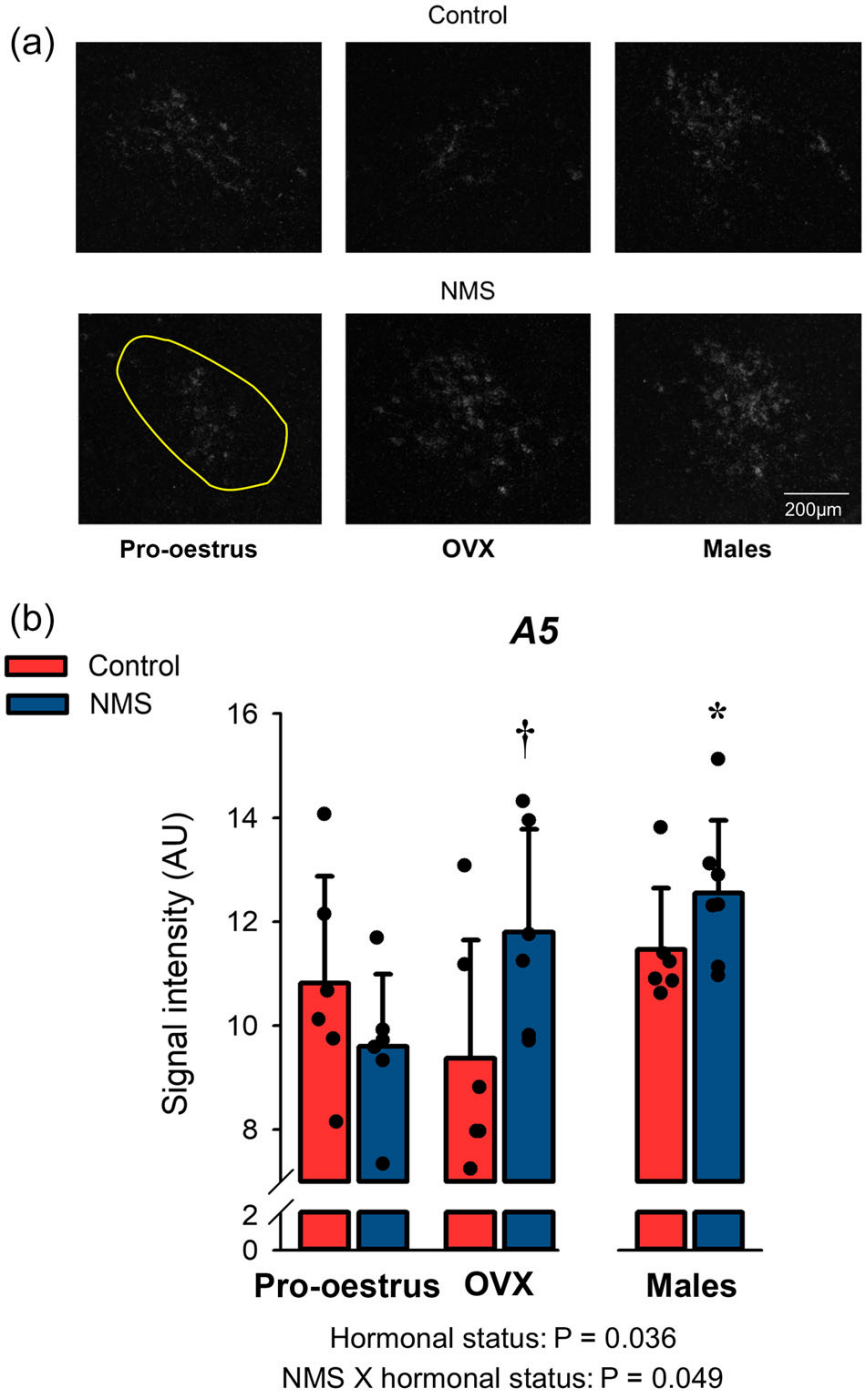
Effects of hormonal status and NMS on OX1‐R expression in the noradrenergic A5 area. (a) Representative photomicrographs comparing the intensity of the OX1‐R mRNA signal obtained by in situ hybridisation in the A5 area. The yellow area indicates the region analysed in each image; it was not repeated on all panels for simplicity. (b) In this graph, bars represent the mean ± SD; each data point represents the value from a single animal. **†**Different from corresponding control value at *P* < 0.05. *Different from corresponding pro‐oestrus value at *P* < 0.05. NMS, neonatal maternal separation; OX1‐R, orexin‐1 receptor.

Here, NMS influenced the effect of OVX on OX1‐R expression; following OVX, the intensity observed in NMS was 26% greater than controls (Figure 6). Moreover, the signal was 18% more intense in males than in pro‐oestrus; this effect was most noticeable in the NMS group (Figure 6; Table 2).

### 3.5 Hormonal status influences plasma levels of ORX_A_

**FIGURE 7:**
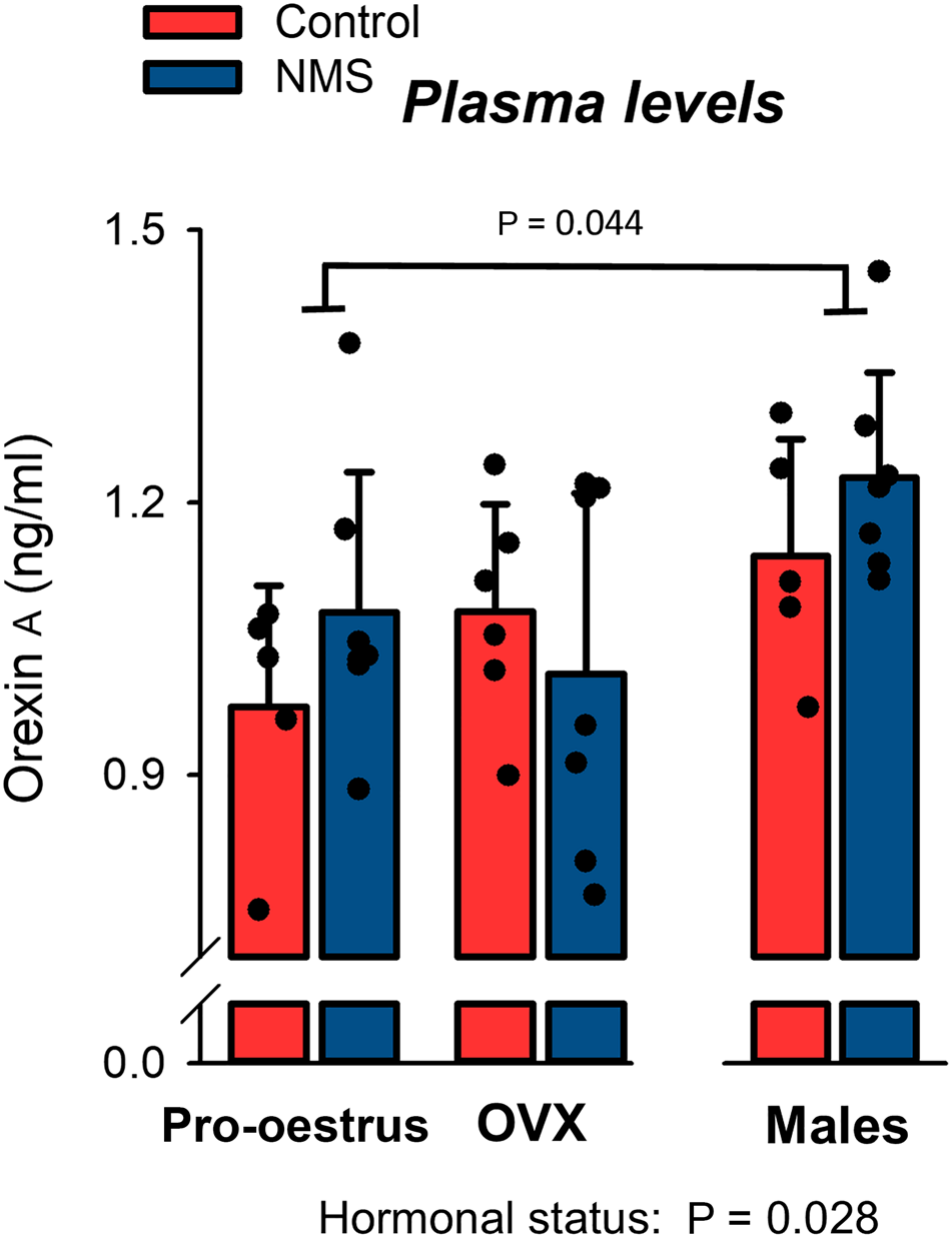
Effects of hormonal status and NMS on plasma levels of orexin A. Bars represent the mean ± SD; each data point represents the value from a single animal. NMS, neonatal maternal separation.

The lowest ORX_A_ values were observed in the group with the highest levels of ovarian hormones (pro‐oestrus), whereas the levels measured in males were 17% higher than pro‐oestrus (Figure 7; Table 2). However, NMS did not influence circulating levels of ORX_A_.

## 4 DISCUSSION

Excessive cardiorespiratory response to CO_2_ inhalation is a diagnostic tool for stress‐related neurological disorders such as PD and PTSD (Sanderson & Wetzler, 1990). Both disorders are more prevalent in women, and anomalies in ORX signalling have emerged as an important pathophysiological mechanism. In females, oestrogens generally inhibit excitatory signals converging onto ORX neurons; however, previous exposure to NMS interferes with this mechanism (Tenorio‐Lopes et al., 2020). Here, we determined whether NMS and hormonal status interact to influence OX1‐R expression. We demonstrate that hormonal status alone has an important influence on the intensity of the OX1‐R signal in the MeA, RObs and A5 area; however, the direction of the changes (increase vs. decrease) varied between structures. We also show that prior exposure to NMS had persistent effects on the way hormonal status modulates OX1‐R expression and body weight. Significant factorial interactions were noted in the DR, the LC, NTS and A5, but such heterogeneity in the effects of hormonal status and NMS was not expected. The DR is the only structure studied in which NMS‐ and sex‐related changes in OX1‐R expression match stress‐induced enhancement of the CO_2_ response. The DR is, therefore, a prime candidate in future mechanistic studies of PD and PTSD.

### 4.1 Critique of methods

While there is still substantial debate as to which specific cells are ‘true CO_2_ sensors’ (Gonye & Bayliss, 2023), it is well established that regulation of the cardiorespiratory responses to CO_2_ involves numerous brain structures, many of which receive significant ORX innervation (Gestreau et al., 2008; Li & Nattie, 2014; Nattie & Li, 2011). The use of in situ hybridisation provided the anatomical resolution necessary to focus our investigation on many (but not all) brain areas that are important in the regulation of the reflexive responses to CO_2_. One notable omission is the retrotrapezoid nucleus (RTN), which currently stands as the primary CO_2_ sensing structure (Guyenet et al., 2019). Although this medullary structure receives ORX innervation (Lazarenko et al., 2011), the small size and diffuse nature of the RTN made it difficult to obtain a robust signal with our approach.

Circadian rhythm has a strong influence on the ORX system, including OX1‐R expression (Silveyra et al., 2009). ORX potentiates the ventilatory response to CO_2_ (Carrive & Kuwaki, 2017; Gestreau et al., 2008; Li & Nattie, 2014; Nattie & Li, 2012); however, the inhibitory action of the selective OX1‐R antagonist SB334867 on the intensity of the ventilatory response to CO_2_ of males is greatest during the dark phase, when rats are active (Dias et al., 2010, 2009). Because tissues were harvested only during the light phase, we could not determine whether circadian rhythm interacts with NMS and/or hormonal status to regulate OX1‐R expression or plasma ORX_A_. That said, substantial differences in SB334867 efficacy at attenuating the CO_2_ response between NMS and controls have been detected in experiments performed on female rats during the light phase (Tenorio‐Lopes et al., 2020). Considering that nightly panic attacks are reported in ∼50% of individuals diagnosed with PD and PTSD (Levitan & Nardi, 2009) and that these patients are ∼2 times more likely to suffer from SDB than the healthy population (Hombali et al., 2018; Lin & Winkelman, 2012), the present work remains highly relevant.

As functional outcomes were not evaluated in the present study, a comparison of body weights between groups confirmed the efficacy of our protocols. Specifically, the higher body weight of OVX females is consistent with a reduction in ovarian function (Mahboobifard et al., 2022); data showing that NMS augmented body weight of males (but not females) is in line with the sex‐specific augmentation of hypothalamo–pituitary–adrenal axis activity (for review, see Tenorio‐Lopes & Kinkead, 2021; van Bodegom et al., 2017), along with our previous work and the clinical literature (Fournier et al., 2015; Torres & Nowson, 2007). Sex hormones were not measured, but we previously showed that in females with intact ovaries, oestradiol peaks during pro‐oestrus and is lowest during metoestrus; OVX reduces circulating levels of progesterone and oestradiol significantly, and the drops were similar in NMS and controls (Fournier et al., 2015; Tenorio‐Lopes et al., 2020). We also reported that in males, basal levels of testosterone, oestradiol and progesterone in NMS rats are similar to those of controls (Fournier et al., 2014). However, the fact that sex hormones were not measured on the same samples as those that were used to quantify plasma ORX_A_ is a limitation of the present work, as it was not possible to determine whether these levels correlate.

### 4.2 Sex‐based differences in OX1‐R expression in the amygdala

The amygdala is composed of multiple subregions and plays a central (and complex) role in the generation of fear and the pathogenesis of anxiety. Furthermore, the amygdala contains neurons that show CO_2_‐sensing properties, and activation of these cells by CO_2_/H^+^ triggers fear‐related responses but not hyperventilation (Ziemann et al., 2009). Such dual roles in the regulation of behavioural versus respiratory responses to CO_2_ inhalation are in line with clinical and pre‐clinical data showing that the amygdala attenuates the ventilatory response to CO_2_ (Dlouhy et al., 2015; Feinstein et al., 2022; Tenorio‐Lopes et al., 2017). On the one hand, our data showing that OX1‐R expression in the MeA is higher in males than females is consistent with the sex‐based difference in the prevalence of PD and PTSD. On the other hand, the lack of difference between pro‐oestrus and OVX contrasts with observations made in the hypothalamus, where oestrogens favour OX1‐R expression. Together, these data do not support the notion that a change in OX1‐R within this structure is an important mechanism in respiratory manifestations of stress‐related neurological disorders. That said, the MeA of rodents contains a large population of aromatase‐expressing neurons (Billing et al., 2020) and stress affects aromatase expression in the brain (Dickens et al., 2013), so this enzyme may dampen the impact of stress within this structure.

### 4.3 NMS interferes with the oestrogen stimulation of OX1‐R expression in the LC

The LC is the main noradrenaline‐producing structure in the brain, and in addition to its contribution to the regulation of arousal states, there is considerable evidence indicating that LC neurons display intrinsic CO_2_ sensitivity (Filosa et al., 2002; Gonye & Bayliss, 2023). Because some authors consider anxiety disorders as states of hypervigilance, LC has been linked to panic attacks and other stress‐related neurological issues (Ross & Van Bockstaele, 2021; Sullivan et al., 1999). The changes in OX1‐R expression observed in control females are in line with previous reports indicating that oestradiol augments OX1‐R expression in the hypothalamus, pituitary and ovaries (Silveyra et al., 2009, 2007), but clearly, NMS interfered with this action by reversing the pattern of expression in low versus high hormonal states. Preclinical studies show that ORX projections to the LC are part of the pathway activating the fear response (Soya & Sakurai, 2020), but to the best of our knowledge, the extent to which this pathway contributes to CO_2_‐induced hyperventilation during a panic‐like event has not been tested specifically. Considering that OVX reduces the CO_2_ response of NMS females but not controls (Tenorio‐Lopes et al., 2020), the increase in OX1‐R expression observed in the LC of OVX + NMS females does not argue for an important role of this pathway in the excessive CO_2_ response observed in stressed females.

### 4.4 OX1‐R expression in serotonin (5‐HT)‐producing structures

Anomalies in the brain serotonergic system play a critical role in the pathophysiology of PD and PTSD (Hale et al., 2012). 5‐HT‐producing neurons are organised in multiple structures in the brainstem and medulla. Although there is evidence indicating that the RObs and DR neurons are CO_2_ sensors, direct measures of CO_2_ effects on raphe firing activity in vivo have yielded inconsistent results (Gonye & Bayliss, 2023). Regardless, the DR projects to the rostral ventrolateral medulla that contains sympatho‐excitatory neurons and the RTN (Bago et al., 2002; Moreira et al., 2015; Vertes & Kocsis, 1994). ORX projections to the DR excite these neurons via both OXR subtypes (Brown et al., 2002; Mavanji et al., 2022), and whereas CO_2_ or optogenetic stimulation of DRN neurons mediates arousal from sleep, it has limited impact on breathing (Kaur et al., 2020; Smith et al., 2018). While it was proposed that the modest increase in breathing following DRN stimulation more likely reflects the change in sleep/wake state than a direct action, we must keep in mind that these experiments were exclusively performed on male mice and that the impacts of stress on the brain are sex‐specific. This is worth considering because early life stress and female sex are significant risk factors for PD and PTSD, and in rats NMS augments the CO_2_ response of females (but not males) especially during pro‐oestrus. We emphasise these points because the DRN is the only structure studied here in which the sex‐specific effects of NMS on OX1‐R expression match the physiological phenotype observed in NMS exposed rats: increased receptor expression following NMS in pro‐oestrus females is consistent with an enhanced sensitivity to a selective OX1‐R antagonist (Tenorio‐Lopes et al., 2020). This would argue that a strengthened excitatory influence of ORX on this population of 5‐HT neurons is an important ‘panic on’ mechanism in the pathophysiology of stress‐related disorder, especially in females during pro‐oestrus. This pathway would balance the ‘panic off’ influence that DR neurons exert on perifornical area where an important population of ORX neurons is located (Bernabe et al., 2024).

By comparison with the DR, RObs innervation of the lower brainstem and spinal cord regions involved in autonomic and respiratory control is more important, as selective photo‐stimulation of the RObs triggers robust cardiorespiratory responses (Bago et al., 2002; DePuy et al., 2011). The RObs also receive significant ORX innervation, and our results showing the intensity of the OX1‐R signal observed in the RObs and DR is consistent with previous reports (Berthoud et al., 2005; Marcus et al., 2001). Expression of steroid‐regulating enzymes, such as aromatase, offers a plausible explanation for region‐specific effects of hormonal status, but to the best of our knowledge, neuroanatomical studies of this enzyme in rodents have been limited to the forebrain (Foidart et al., 1995; Horvath & Wikler, 1999). It is, therefore, difficult for us to explain the different effects of NMS and stress on OX1‐R expression between the two 5‐HT producing structures studied here. In light of the limited impact of hormonal status and the lack of effect of NMS, we conclude that, unlike the DR, the potential contribution of the RObs to cardiorespiratory manifestations of PD and PTSD remains limited.

### 4.5 Sex‐specific effects of NMS on OX1‐R expression in the NTS

Within the medulla, the NTS is the primary projection site for numerous cardiorespiratory afferents (Housley, 1991). It also projects to key CO_2_‐sensing areas, and NTS neurons may themselves have CO_2_‐sensing properties (Dean & Putnam, 2010). While NTS activation typically stimulates cardiorespiratory function, this structure also contains a significant population of GABAergic neurons that can inhibit breathing by acting on medullary neurons, generating respiratory rhythm (Fong et al., 2005; Shao et al., 2024). However, the contribution of NTS‐GABAergic neurons to respiratory control remains limited (Shao et al., 2024). Here, the effects of NMS and hormonal status on OX1‐R expression observed in the NTS are remarkable because they are in opposition to the respiratory phenotype reported in rats subjected to NMS. For instance, while attenuation of the CO_2_ response following OVX was greater in NMS females than controls (Tenorio‐Lopes et al., 2020), our data show paradoxically increased OR1‐R expression in the NTS of OVX + NMS females. While we do not know the phenotype of OX1‐R expressing cells in the NTS, it is tempting to propose that their activation inhibits the cardiorespiratory response to CO_2_.

### 4.6 Sex‐specific effects of NMS on OX1‐R expression in the A5 area

Mainly composed of catecholaminergic neurons, the A5 region has multiple connections with the amygdala along with medullary structures regulating cardiorespiratory function. It is of great interest here as electrical stimulation of the ‘hypothalamic defence area’, a region rich in ORX neurons, activates A5 neurons via direct projections (López‐González et al., 2013). Our main observation in this structure is that OVX augments OX1‐Rs in NMS but not controls and that the highest levels were achieved in males. Much like the NTS, these results generally oppose the sex‐specific effects of NMS on the CO_2_ response and, therefore, suggest that the A5 area plays a limited role in stress‐related cardiorespiratory dysfunction.

### 4.7 Regulation of circulating ORX_A_ levels

Change in the capacity of ORX production has numerous impacts on health. Narcolepsy is perhaps the best example as ORX deficiency causes this disease; reduced ORX_A_ in the CSF is a definitive criterion for diagnosis (Barateau et al., 2023; Mignot et al., 2002). Of note, ORX_A_ levels are also reduced in CSF samples of PTSD patients (Strawn et al., 2010). ORXs are exclusively produced by hypothalamic neurons but because ORX_A_ is a lipophilic peptide that crosses the blood–brain barrier more easily and it is less rapidly metabolised than ORX_B_ , plasma levels can be measured more easily (Kastin & Akerstrom, 1999). By comparison with lumbar puncture, measuring plasma ORX_A_ is less invasive but the difference between healthy versus narcoleptic or PTSD subjects is significantly less that observed in CSF samples (Mignot et al., 2002; Strawn et al., 2010; Zhu et al., 2020). Conversely, comparison of plasma ORX_A_ between patients with sleep disordered breathing and healthy subjects has yielded highly variable results and the value of plasma ORX_A_ as a biomarker for this disorder is uncertain (Igarashi et al., 2003; Mohammadi et al., 2023; Sakurai et al., 2005). Having previously shown that ORX_A_ levels in hypothalamic extracts of NMS females is 51% higher than controls (Tenorio‐Lopes et al., 2020), we sought to determine whether quantification of plasma levels could detect such stress‐related changes in ORX production. Clearly, the results reported in Figure 7 do not indicate that the between‐group differences measured in hypothalamic extracts can be detected in the plasma. Considering that the same ELISA has been used to quantify brain extracts and plasma and that technical care was taken to optimise sample quality and data variability (sample reservation, collection time), the factors explaining this limitation remain unknown.

### 4.8 Conclusion, future directions and closing comments

Owing to their efficiency at inactivating wake‐promoting systems, dual orexin receptor antagonists (DORAs) offer a new approach to the treatment for insomnia (Hoyer et al., 2020); however, their impacts on other neurological comorbidities show significant inter‐ and intra‐individual variability that compromises a clear conclusion on the therapeutic value of DORAs beyond insomnia (Carpi et al., 2024; Kario et al., 2019; Sun et al., 2016). By demonstrating how hormonal status and NMS influence OX1‐R expression in various brain regions, the present study provides a plausible explanation for the variability in the efficacy of OX1‐R‐acting drugs. While our results show that the DR is a highly promising target for OX1‐R acting agents, it also revealed an important lack of knowledge with regard to the region‐specific mechanism regulating the actions of sex hormones on OX1‐R expression. A definitive anatomical map of regional aromatase‐expressing regions in the brainstem regions would be important progress in that regard.

## AUTHOR CONTRIBUTIONS

Stéphanie Fournier contributed to experimental design, performed the experiments, analysed the data, and contributed to manuscript writing. Julie Plamondon assisted with the experiments, provided technical support, and contributed to manuscript writing. Denis Richard contributed to the conception of the project and assisted with obtaining research funds. As he passed away recently, this author could not contribute to manuscript writing. Richard Kinkead contributed to the conception of the project, obtained research funds, assisted with data analysis, and contributed to manuscript writing. All authors have read and approved the final version of this manuscript and agree to be accountable for all aspects of the work in ensuring that questions related to the accuracy or integrity of any part of the work are appropriately investigated and resolved. All persons designated as authors qualify for authorship, and all those who qualify for authorship are listed.

## CONFLICT OF INTEREST

None declared.

## Data Availability

All data supporting the results are reported in the manuscript. Raw results can be provided upon request.
